# Associations Between Forced Sexual Initiation, Post-exposure Prophylaxis Cascades and Subsequent Violence Experiences Among Displaced Young Women in Ugandan Informal Urban Settlements

**DOI:** 10.1007/s10461-025-04837-1

**Published:** 2025-08-23

**Authors:** Moses Okumu, Carmen H. Logie, Thabani Nyoni, Flora Cohen, Bernadette K. Ombayo, Joseph C. Wabwire, Catherine N. Nafula, Robert Hakiza, Peter Kyambadde

**Affiliations:** 1 School of Social Work, University of Illinois, Urbana-Champaign, 1010 W. Nevada St., Urbana, IL 61801 USA; 2 School of Social Sciences, Uganda Christian University, Mukono, Uganda; 3 Factor Inwentash Faculty of Social Work, University of Toronto, 246 Bloor St. W., Toronto, ON M5S 1V4 Canada; 4 United Nations University Institute for Water, Environment, and Health (UNU-INWEH), 204-175 Longwood Rd. S., Hamilton, ON L8P 0A1 Canada; 5 Faculty of Health, School of Social Work, Dalhousie University, Halifax, Canada; 6 School of Social Work, University of Memphis, 122 McCord Hall, Memphis, TN 38152 USA; 7 Department of Health and Kinesiology, College of Applied Health Sciences, University of Illinois, Urbana-Champaign, Urbana, USA; 8 AVSI Foundation, Plot 16 A, Circular Road, Arua, Uganda; 9 Young African Refugees for Integral Development (YARID), Nsambya Gogonya, Kampala, Uganda; 10 AIDS Control Program, Ministry of Health, Plot 6, Lourdel Road, Nakasero, Kampala, Uganda; 11 Most At Risk Populations Initiative, Kampala, Uganda

**Keywords:** Forced sexual initiation, Intimate partner violence, Non-partner violence, Post-exposure prophylaxis, Displaced youth, Uganda, Iniciación sexual forzada, Violencia de pareja íntima, Violencia por parte de no parejas, Profilaxis posexposición, Juventud desplazada, Uganda

## Abstract

Along their displacement trajectory, displaced adolescent girls and young women (AGYW) face elevated HIV risk early in their sexual life course, often due to forced sexual initiation (FSI), marking the beginning of cycles of violence. However, knowledge gaps exist regarding FSI prevalence rates and the association between FSI, violence experiences, and post-exposure prophylaxis (PEP) cascades (awareness, access and uptake) among displaced AGYW in Uganda. Using peer-driven sampling, we conducted a community-based cross-sectional survey of 201 sexually active displaced AGYW living in informal settlements in Kampala. We conducted bivariate analyses to examine associations between FSI and PEP cascades and multivariable logistic regressions to examine associations between FSI and (a) non-partner physical/sexual violence and (b) recent sexual/physical intimate partner violence (IPV). Among participants (*n* = 72), 35.8% reported forced sexual initiation (FSI); of these, 66.7% experienced lifetime non-partner sexual violence, 81.9% non-partner physical violence, 35.2% recent intimate partner physical violence, and 70.4% recent intimate partner sexual violence. Very few participants who experienced FSI reported awareness of and knowledge of access to PEP in their community, and none had accessed PEP in the past 3 months. Multivariable logistic regression findings showed that compared to AGYW who did not experience FSI, those who experienced FSI had increased odds of reporting non-partner lifetime physical violence, non-partner lifetime sexual violence, intimate partner physical violence, and intimate partner sexual violence. FSI appears to be prevalent among displaced AGYW and is linked to multiple forms of violence, and limited PEP awareness, access and use. Tailored, trauma-informed, multisectoral interventions are needed to address FSI and violence and improve PEP access.

## Resumen

A lo largo de su trayectoria de desplazamiento, las adolescentes y mujeres jóvenes (AGYW) desplazadas enfrentan un riesgo elevado de VIH desde etapas tempranas de su vida sexual, a menudo debido a la iniciación sexual forzada (FSI), lo que marca el inicio de ciclos de violencia. Sin embargo, existen vacíos de conocimiento sobre la prevalencia de FSI y sus asociaciones con experiencias de violencia y el uso de profilaxis posexposición (PEP) cascadas (concienciación, conocimiento del acceso y acceso) entre AGYW desplazadas en Uganda. Realizamos una encuesta transversal basada en la comunidad con 201 AGYW sexualmente activas que viven en asentamientos informales de Kampala, utilizando un muestreo impulsado por pares. Se realizaron análisis bivariados para examinar las asociaciones entre FSI y PEP cascadas, y regresiones logísticas multivariadas para explorar las asociaciones entre FSI y (a) violencia física/sexual por parte de no parejas y (b) violencia física/sexual reciente por parte de parejas íntimas (IPV). Entre las participantes (*n* = 72), el 35.8% reportó FSI; de estas, el 66.7% experimentó violencia sexual por parte de no parejas, el 81.9% violencia física por parte de no parejas, el 35.2% violencia física reciente por parte de parejas íntimas, y el 70.4% violencia sexual reciente por parte de parejas íntimas. Muy pocas participantes que experimentaron FSI reportaron conocimiento o acceso a la PEP en su comunidad; ninguna la había utilizado en los últimos tres meses. Los resultados de las regresiones logísticas multivariadas mostraron que, en comparación con las AGYW que no experimentaron FSI, aquellas que sí lo hicieron tenían mayores probabilidades de reportar violencia física y sexual por parte de no parejas, así como violencia física y sexual por parte de parejas íntimas. La FSI parece ser prevalente entre AGYW desplazadas y está vinculada a múltiples formas de violencia, así como a un conocimiento y uso limitado de la PEP. Se necesitan intervenciones multisectoriales, adaptadas, informadas por el trauma, que aborden la FSI y la violencia, y mejoren el acceso a la PEP.

## Background

Forced sexual initiation (FSI)—a form of sexual violence that occurs when a person’s first sexual encounter is physically forced, pressured, or coerced—remains an intractable public health concern affecting many adolescent girls and young women (AGYW) globally [1–3]. FSI violates AGYW’s reproductive justice of sexual (i.e., inhibiting their ability to consent to or refuse sexual activities) and reproductive (i.e., obstructing them from making informed decisions regarding contraception, pregnancy, and sexually transmitted infection [STI] prevention) autonomy [1, 4, 5]. Experiences of FSI not only breach AGYW’s bodily integrity, but also produce far-reaching adverse mental and sexual health consequences, such as negative feelings or anxiety during sexual activity [4, 6, 7], higher teenage pregnancy rates [8], adverse pregnancy outcomes [9], and a long-term risk of STIs [7, 10, 11], including HIV [12]. Whether viewed in the short or long term, FSI can impair AGYW’s ability to exercise agency in future sexual and reproductive choices, which may result in adverse physical and mental health repercussions.

A limited number of studies have examined the relationship between FSI vulnerability and violence experiences. For example, a study involving youth (aged 13–24 years) in Nigeria, Uganda, and Zambia found that FSI was associated with violent experiences, inconsistent condom use, and mental health challenges [13]. For AGYW, Michau et al. ’s [14] framework indicates that harmful gender norms contribute to or exacerbate experiences of both FSI and future violence at the individual, interpersonal, community, and societal levels [14]. At the *individual* level, AGYW may internalize and reproduce inequitable and harmful gender norms by defending and remaining in abusive relationships. Violence against AGYW largely occurs in *interpersonal* interactions, especially in family contexts (often where AGYW first encounter harmful gender norms regarding women and girls), which can shape their attitudes and behaviors regarding violence. At the *community* level, these harmful gender norms can cause AGYW who experience violence to be shamed or prevented from reporting, leaving these norms unaddressed and resulting in cycles of violence. At the *societal* level, societal and institutional laws, policies, and service infrastructure shape public understanding and practical responses to violence against AGYW. For instance, inequitable gender norms prevent displaced youth from accessing post-rape clinical care services (e.g., post-exposure prophylaxis [PEP] and emergency contraception), especially when they are not sufficiently informed about these services [15, 16]. While PEP is effective in preventing HIV infection among sexual violence survivors [17], knowledge gaps persist regarding rates of awareness, access, and uptake, particularly among displaced adolescents in African contexts [18]. Given AGYW’s documented vulnerability to both FSI and violence at multiple ecological levels, investigating the relationship between these two types of vulnerabilities is essential for effectively designing and distributing interventions and resources that target displaced AGYW.

The need for FSI prevention programming for forcibly displaced AGYW (e.g., due to armed conflict, persecution, or political instability) [19] is particularly pronounced in Africa, which in 2024 housed approximately 80% of the world’s 120 million forcibly displaced persons, most of whom were girls or women [20]. Furthermore, a recent study spanning nine African countries found that even among *non*-forcibly displaced AGYW (13–24 years of age), the FSI prevalence ranged from 14.7 to 38.9%, and the rates of FSI among forcibly displaced AGYW in those countries may be even higher [5]. Given the evidence that experience of FSI (whether before, during, or after displacement) usually marks the beginning of an ongoing cycle of violence [19, 21, 22], Africa’s forcibly displaced AGYW represent a substantial and highly vulnerable population who stand to benefit in both the short- and long-term from FSI prevention programming and resources. AGYW who are forcibly displaced from their home communities are particularly likely to face a continuum of violence that extends beyond the displacement journey and into resettlement. The heightened physical and psychological vulnerability of AGYW in these contexts may further increase their likelihood of experiencing FSI and, in turn, increase their vulnerability to acquiring HIV [19].

Uganda presents an ideal setting for studying associations between FSI, PEP cascades and violence experiences among forcibly displaced AGYW. The number of new HIV-positive diagnoses in Uganda has decreased in the general population from 94,000 in 2010 to 38,000 in 2020, marking a 60% reduction [23]. However, the weekly number of 1,100 new HIV-positive persons—of whom 64% are girls or women—is still alarmingly high and needs urgent redress [23]. Uganda is home to more than 1.8 million refugees [24] and has an estimated 15.2% prevalence of FSI among all girls and young women (13–24 years old) nationally [25]. With many forcibly displaced youth preferring to move to Uganda’s urban centers to pursue economic opportunities, Uganda’s capital, Kampala, hosts over 116,000 forcibly displaced persons, 27% of whom are aged 15–24 years [24]. Many of these displaced persons in Kampala also live in informal settlements, including slums [26–29], and experience poverty, overcrowding, violence, depression, and disproportionately high HIV prevalence [30–35], all of which increase their vulnerability to FSI. A 2019 study among forcibly displaced AGYW in Kampala also found that experiences of multiple forms of violence during childhood were associated with an increased likelihood of experiencing subsequent non-partner violence [30]. Additionally, several studies conducted among AGYW in different African countries with *non-displaced* persons have identified FSI as a significant risk factor for HIV acquisition [36, 37]. Unfortunately, while early prevention efforts are critical for reducing vulnerability to FSI and other forms of sexual violence over the life course, interventionists and policymakers lack sufficient evidence on FSI among displaced AGYW to design effective trauma-informed violence-prevention interventions for this population.

Despite growing evidence of (a) links between FSI and sexual and reproductive health outcomes (including exposure to HIV) and (b) AGYW’s vulnerability to experiences of violence and FSI, no study has assessed the rates of FSI among displaced AGYW in Uganda or FSI’s association with PEP cascades or future experiences of violence. Guided by Michau et al. ’s [14] framework for assessing the causes of violence against AGYW at multiple ecological levels, the present study used a convenience sample to address three research questions focused on displaced AGYW in urban Kampala, Uganda: (1) At the individual level, what is the prevalence of FSI? (2) At the interpersonal level, are there any associations between FSI and (a) non-partner violence and (b) recent intimate partner violence?; and (3) At the structural level, is FSI associated with PEP knowledge, availability, and utilization?

## Methods

We implemented our community-based cross-sectional study in collaboration with both refugee-led community-based agencies and government agencies from January 2018 to April 2018 in Kampala, Uganda. Eligible participants included young women aged 16–24 years who reported (a) being a refugee or displaced person or having refugee/displaced parents, (b) living in an informal settlement (i.e., Kabalagala, Kansanga, Katwe, Nsambya, or Rubaga), and (c) being able to provide informed consent. We recruited and trained eight refugee-identified young women aged 18–24 years as peer research assistants (PRAs).

PRAs recruited participants using convenience sampling methods, including peer outreach and word of mouth. PRAs provided each participant with a recruitment flyer that included their contact information and invited them to recruit up to five refugee/displaced youth from their social networks. PRAs administered a tablet-based survey at a location chosen by the participant (e.g., football pitch or community agency). PRAs ensured that participants had privacy as they completed the 35- to 45-minute tablet-based survey. Each participant received a 12,500 UGX (approximately $3.74) honorarium for completing the survey. The study protocol was approved by the University of Toronto and the Uganda Ministry of Health (ADM: 105/261/01). For this study, a subsample of only those participants who reported having engaged in sexual intercourse were considered for the analysis.

### Measures

*The outcome variables* included two types of violence: (1) non-partner lifetime violence [38, 39] (i.e., violence perpetrated by anyone other than an intimate partner, whether another family member, friend, acquaintance, neighbor, or stranger) experienced after age 16, and (2) intimate partner violence (IPV) experienced in the last 12 months (i.e., recent sexual and physical IPV). To assess non-partner violence, participants were asked: “*Have you ever experienced at age 16 years old and over (check all that apply); sexual violence (yes = 1*,* no = 0); physical violence (yes = 1*,* no = 0)*.” IPV was assessed using IPV questions from the Brief Inpatient Screen for IPV [40]; these questions were only completed by participants who were currently in an intimate relationship or had been in the past 12 months prior to the survey. The questions assessed physical and sexual IPV in the last 12 months. For instance, physical violence was assessed with the question: “*During the past 12 months, how many times did someone you were dating physically hurt you on purpose (such as hit*,* slammed you into something)?*”; and sexual violence was assessed with the question, “*During the past 12 months, how many times did someone you were dating force you to do sexual things you did not want to do (such as kissing*,* sex)*?” Response options included: Never, 1 time, 2 or 3 times, 4 or 5 times, and 6 or more times. For both physical and sexual IPV, responses were categorized as no violence = 0 or experienced violence = 1. The use of simpler measures of non-partner and IPV is a limitation of our study as our estimates of exposure to physical/sexual violence may be under-reported, since the multi-items questions aren’t asked. We used the simpler measure because the study was designed as an HIV study, and we were careful not to increase participation burden.

The other outcome variables included PEP awareness, access, and uptake. Surveys assessed PEP availability and awareness of PEP availability [41], by asking participants whether “*Post-exposure prophylaxis (HIV medication taken 72 hours after unprotected sex to avoid becoming HIV infected) is available in my area (yes = 1*,* no = 0).*” We assessed participants’ knowledge of PEP access by asking them whether “*I know how to access PEP (yes = 1*,* no = 0).*” We assessed participants’ recent PEP uptake by asking them whether “*I have accessed PEP in the last 3 months (yes = 1*,* no = 0).*”

The *independent variable* of interest was *forced sexual initiation (FSI; yes = 1*,* no = 0)*, assessed by asking whether participants were forced during their first experience of sexual intercourse. An affirmative response was coded as an FSI experience.

*Covariates* included *sociodemographic variables*: age (continuous), education level (dichotomous: no formal education/less than secondary school vs. post-secondary education and above), time living in Uganda (< 5 years, 6–10 years, >10 years), employment status (dichotomous: employed/student vs. unemployed), and family structure (categorical: living with one parent, living with both parents, or living with neither parent).

#### Statistical Analysis

We first conducted descriptive analyses of all variables to determine the frequencies and proportions. Descriptive analyses included all participants with available data from the study. First, we conducted bivariate analyses (i.e., *t-test*, *χ²*, Fisher’s exact test for PEP variables) to determine differences by forced first sexual intercourse. Second, we conducted multivariable logistic regression to examine the association between violence experiences and forced first sexual intercourse, adjusting for sociodemographic factors (i.e., age, education, employment, and family structure). We then identified the adjusted odds ratios (aORs) for the multivariable logistic regression, highlighting those that were significant at the *p* < 0.05 level. Missing responses were excluded from the analyses, and the number of complete responses was reported for each variable. All statistical analyses were performed using Stata version 14.

## Results

Among a convenience sample of 201 AGYW, 35.8% (*n* = 72) reported experiencing FSI. Among AGYW who reported experiencing FSI, 72.2% (*n* = 52/72) had an early sexual debut (i.e., sex before the age of 15 years), 59.7% (*n* = 43/72) did not have a secondary school education, and almost half (*n* = 35/72) were unemployed and not attending school. Regarding post-FSI experiences of violence, 66.7% (*n* = 48/72) had experienced lifetime non-partner sexual violence, 81.9% (*n* = 59/72) had experienced lifetime non-partner physical violence, 35.2% (*n* = 19/46) had experienced recent intimate partner physical violence, and 70.4% (*n* = 38/54) had experienced recent intimate partner sexual violence (Table 1).

**Table 1:** Factors associated with forced sexual initiation among displaced AGYW living in informal urban settlement in Kampala, Uganda (*N* = 201)

Indicators	Total *N* (%)/Mean (SD)	Ever forced at first sex (*n* = 72; 35.8%) *N* (%)/Mean (SD)	Never forced at first sex (*n* = 129; 64.2%) *N* (%)/Mean (SD)	*p*-value
Age^a^	20.28 (2.53)	19.14 (2.45)	19.60 (2.59)	0.222
Education				**0.015**
*>Secondary school*	97 (48.3)	43 (59.7)	54 (41.9)	
*Secondary school*	104 (51.7)	29 (40.3)	75 (58.1)	
Time in Uganda				0.543
*< 5 years*	135 (67.1)	51 (70.8)	84 (65.1)	
*6–10 years*	53 (26.4)	18 (25.0)	35 (27.1)	
*>10 years*	13 (6.5)	3 (4.2)	10 (7.8)	
Family Structure				0.285
*Living with both parents*	64 (32.2)	22 (31.0)	42 (32.8)	
*Living with one parent*	44 (22.1)	19 (26.8)	25 (19.5)	
*Living with neither parent*	91 (45.7)	30 (42.3)	61 (47.7)	
Current Employment				**0.0001**
*Student*	59 (29.3)	10 (13.9)	49 (38.0)	
*Employed/Student*	44 (21.9)	27 (37.5)	17 (13.2)	
*Unemployed*	98 (48.8)	35 (48.6)	63 (48.8)	
Early Sexual Debut				**0.0001**
*< 15 years*	104 (51.7)	20 (27.8)	84 (65.1)	
*>15 years*	97 (48.3)	52 (72.2)	45 (34.9)	
**Violence Experiences**				
Lifetime Non-partner Physical Violence^b^				**0.006**
*No*	60 (29.9)	13 (18.1)	47 (36.4)	
*Yes*	141 (70.1)	59 (81.9)	82 (63.6)	
Lifetime Non-partner Sexual Violence^b^				**0.0001**
*No*	128 (63.7)	24 (33.3)	104 (80.6)	
*Yes*	73 (36.3)	48 (66.7)	25 (19.4)	
Recent Physical IPV^c^ (*n* = 173)				0.085
*No*	127 (73.4)	35 (64.8)	92 (77.3)	
*Yes*	46 (26.6)	19 (35.2)	27 (22.7)	
Recent Sexual IPV^c^ (*n* = 173)				**0.004**
*No*	79 (45.7)	16 (29.6)	63 (52.9)	
*Yes*	94 (54.3)	38 (70.4)	56 (47.1)	

^a^Independent *t*-test; ^b^“lifetime” indicates violence experienced after age 16; ^c^“recent” indicates violence experienced in the last 12 months; ^d^Fisher’s exact test.

### Associations Between FSI and PEP Awareness, Knowledge, and Recent Access

**Fig. 1:**
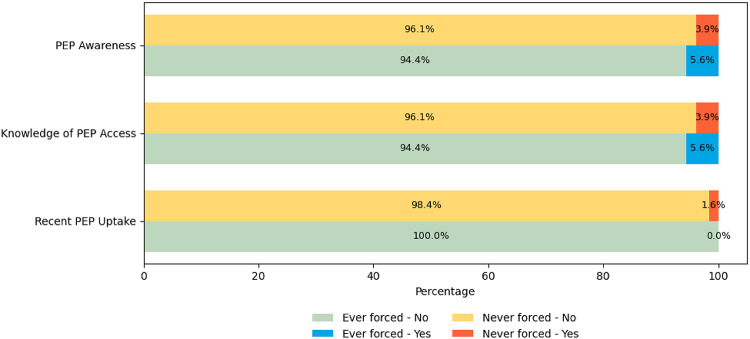
Forced sexual initiation and PEP awareness, knowledge of access, and recent access

Using chi-square independence tests (Table 1), we found no statistically significant differences in PEP cascades among participants by FSI. We found that across the entire sample, only 4.5% (*n* = 9/201) reported PEP awareness and knowledge, and 1% (*n* = 2/201) reported PEP uptake. Among participants who experienced FSI, 5.6% (*n* = 4/72) reported awareness of community PEP resources, 5.6% (*n* = 4/72) reported knowing where to access PEP, and none reported accessing PEP in the past 3 months (see Fig. 1).

### Associations Between FSI and Violence Experiences Among Urban Forcibly Displaced AGYW

In the adjusted analyses, AGYW who experienced FSI reported increased odds of non-partner lifetime physical violence (aOR = 2.88; 95% CI = [1.33, 6.22]), non-partner lifetime sexual violence (aOR = 13.22; 95% CI = [5.85, 29.88]), recent intimate partner physical violence (aOR = 2.56; 95% CI = [1.12, 5.88]), and recent intimate partner sexual violence (aOR = 2.31; 95% CI = [1.02, 5.20]) compared to AGYW who did not experience FSI (Table 2).

**Table 2:** Associations between forced sexual initiation and violence experiences among displaced urban AGYW in kampala, Uganda (*N* = 199)

	Lifetime Physical Violence			Lifetime Sexual Violence			Physical IPV			Sexual IPV		
	aOR	95% CI	*p*-value	aOR	95% CI	*p*-value	aOR	95% CI	*p*-value	aOR	95% CI	*p*-value
Forced first sexual intercourse vs. No forced first sexual intercourse												
No forced first sexual intercourse (Ref) Yes forced first sexual intercourse	2.88	1.33–6.22	**0.007**	13.22	5.85–29.88	**0.001**	2.56	1.12–5.88	**0.027**	2.31	1.02–5.20	**0.044**
Age	0.99	0.85–1.16	0.898	0.92	0.78–1.091	0.343	0.78	0.65–0.95	0.012	0.88	0.74–1.04	0.125
Education (Ref *> secondary school*)	1.53	0.73–3.18	0.26	1.41	0.64–3.05	0.387	4.14	1.72–9.96	**0.001**	1.22	0.57–2.56	0.622
Employment (Ref *employed*)												
Student	0.95	0.33–2.79	0.928	1.69	0.5–5.72	0.396	0.54	0.17–1.76	0.310	0.16	0.05–0.49	**0.001**
Unemployed	0.92	0.36–2.29	0.855	3.64	1.32–10.07	**0.013**	1.32	0.50–3.45	0.574	0.57	0.23–1.48	0.252
Family structure (Ref *living with both parents*)												
Living with one parent	0.64	0.26–1.53	0.31	0.62	0.24–1.62	0.329	0.53	0.19–1.48	0.226	1.00	0.39–2.53	1.00
Living with neither parent	0.92	0.44–1.92	0.823	0.62	0.28–1.38	0.242	0.57	0.25–1.35	0.204	3.53	1.56–7.94	**0.002**

## Discussion

As many forcibly displaced AGYW experience FSI (either before, during, or after displacement) while facing subsequent and overlapping traumatic experiences, researchers, interventionists, and policymakers must develop effective interventions that prevent future violence and, in turn, STIs among this vulnerable population [13, 19, 21, 22]. Guided by Michau et al. ’s [14] framework, our study examined the prevalence of FSI among displaced AGYW and its association with (a) non-partner violence and recent IPV and (b) PEP cascades (knowledge, availability, and utilization).

Our study found that more than a third of reproductive-age displaced AGYW in our sample experienced FSI, more than double Uganda’s national FSI prevalence rate (15%) and the FSI rates observed in studies of non-displaced youth (14–18%) [11, 42]. This alarmingly high prevalence of FSI observed among urban displaced AGYW is particularly concerning, given that other studies [11, 25, 42] conducted among *non-*displaced AGYW in different African countries have identified FSI as a significant risk factor for HIV acquisition [31]. Similarly, experiencing FSI may undermine the sexual and reproductive autonomy of AGYW survivors by taking away their right to decide whether and when to initiate sexual relations [4]. The increased prevalence of FSI among this population can be attributed to several factors, including economic insecurity, limited access to protective services, and the breakdown of traditional social support systems that often occur during displacement. Furthermore, living in informal urban settlements may exacerbate these risk factors, as displaced AGYW may have to navigate unfamiliar environments with potentially reduced community oversight and increased exposure to exploitation and abuse. The high rate of FSI in our sample underscores the urgent need for targeted interventions and support services specifically designed to address the unique challenges faced by urban displaced AGYW in Uganda. These interventions could aim to improve AGYW’s knowledge, access to post-exposure prophylaxis (PEP), and comprehensive sexual and reproductive health education.

Our analyses also found that FSI was associated with experiences of non-partner and intimate partner physical and sexual violence, which are key indicators of HIV vulnerability. These findings corroborate prior evidence of FSI’s association with multiple types of recent violence in various global contexts [5, 11–13] and among displaced AGYW living in Kampala [30]. Michau and colleagues’ [14] framework posits that the complex interplay between FSI and violences experiences span ecological levels. For instance, at the individual level, overlapping experiences of FSI and displacement may amplify AGYW’s interpersonal trauma and insecurity in ways that increase their likelihood of experiencing other forms of violence beyond FSI (i.e., partner and non-partner violence). Specifically, many AGYW who experience FSI may also experience other forms of violence from the same perpetrator [11]. These AGYW may normalize violence in intimate partnerships, influencing how they defend themselves or negotiate safer sexual practices in the future. For instance, choices such as remaining in abusive relationships may increase the risk of violence and its associated adverse outcomes (e.g., STIs, including HIV). Thus, interventions seeking to sustainably address personal trauma and promote healthy relationships among FSI-affected displaced AGYW may consider (a) engaging both men and women in critical dialogues on the existence, causes, and consequences of FSI and (b) developing targeted support for displaced AGYW who have experienced FSI, given this population’s elevated vulnerability to multiple forms of violence and associated negative health outcomes.

On a more granular level, our results show the association between AGYW’s experiences of FSI and their knowledge of, access to, and utilization of post-rape care services [14], such as PEP [15]. Among participants who experienced FSI, only 5.6% were aware of PEP’s availability or knew where to access it in their community, and *none* had accessed PEP in the past 3 months. While ours is the first Africa-based study to assess the levels of knowledge of availability, access, and utilization of PEP among FSI survivors, our findings align with prior evidence that refugee and displaced young women (ages 16–24 years) have limited knowledge of available biomedical interventions (e.g., pre-exposure prophylaxis) [15, 43]. Along with others [15, 16], we suggest that the normalization of silence and acceptance of FSI against AGYW may contribute to this population’s insufficient knowledge of the existence, location, and purpose of PEP services. This gap in knowledge can have further repercussions for displaced AGYW. Experiencing FSI places AGYW at an elevated risk of contracting HIV and other sexually transmitted infections [5, 11–13]. Moreover, AGYW carry a disproportionate burden of the HIV epidemic globally [44, 45]. To mitigate this risk, displaced AGYW who have experienced FSI need trauma-informed PEP interventions delivered in local languages and youth-friendly ways to promote their engagement with and uptake of PEP [15]. These trainings may also enhance providers’ ability to screen survivors of violence for any forced or coercive first sexual initiation and provide relevant education, initiate PEP, and refer them to other services, including counseling and legal redress.

### Implications for Multi-component Interventions Addressing FSI, Violence, and PEP

These findings provide preliminary evidence to inform multi-component interventions spanning socio-ecological levels. At the individual and interpersonal levels, implementing peer education programs and bystander intervention training using participatory comic books [15, 18] could increase awareness of FSI and other prevalent forms of violence and empower youth to recognize and respond to potentially violent situations [15, 30]. Community-level interventions, such as SASA [14]! could engage local leaders in awareness campaigns to help reduce stigma and promote supportive attitudes towards FSI and violence survivors. As structural changes are crucial for improving PEP awareness, access, and utilization, healthcare providers should receive training in youth-friendly, trauma-informed care to create a more welcoming environment for youth survivors seeking help [18, 30]. For instance, significantly improving timely access to post-rape care will require policy amendments to provide around-the-clock PEP and clear referral pathways between community organizations, pharmacies, police, and healthcare facilities.

The necessary work ahead, however, entails not only increasing the availability of and streamlining PEP services, but also increasing displaced AGYW’s ability and willingness to engage with these services. Despite the availability of PEP through the United States President’s Emergency Plan for AIDS Relief (PEPFAR)-supported Determined, Resilient, Empowered, AIDS-free, Mentored, and Safe (DREAMS) program [46], the initiative has primarily focused on non-displaced AGYW, resulting in heightened awareness and access within this group. Conversely, a significant number of displaced AGYW in Uganda remain uninformed about PEP and its accessibility, leading to persistent deficiencies in knowledge and service utilization. This existing disparity is particularly alarming given the current uncertainty regarding future PEPFAR funding under the Trump administration, which poses a threat to further diminish HIV prevention resources. Should such funding become restricted or unstable, efforts to reach displaced AGYW are likely to be deprioritized or eliminated altogether, thereby exacerbating inequities in PEP access [47]. To address these increasing disparities, it is imperative to implement urgent, sustained, and targeted educational and service delivery initiatives to ensure that displaced AGYW, who are among the most vulnerable populations, are not marginalized. Efforts to overcome access barriers to PEP initiation may also entail following the World Health Organization’s recommendation that governments introduce and expand PEP-in-pocket initiatives, in which survivors and those at increased risk receive emergency PEP kits for immediate use [17]. Ultimately, governments should consider integrating PEP more broadly into HIV prevention services and transition program management to local partners to reduce inequities and increase access to all. Future research should focus on evaluating the effectiveness of these interventions, including innovative approaches such as PEP-in-pocket, and exploring innovative approaches to overcome the unique challenges of access and uptake faced in humanitarian settings.

### Limitations

Despite our study’s potential utility in the future design of gender-transformative interventions that address FSI, experiences of violence, and PEP access and utilization among displaced AGYW, our findings should be interpreted in light of several limitations. First, although using non-probability sampling to recruit participants allowed us to work with our partner community-based organizations to identify the most vulnerable participants to include in our study, our sample may have limited the generalizability of our findings to more than 1.8 million refugees in Uganda and 120 million displaced persons globally. However, our study provides preliminary evidence of the prevalence of FSI and its associations with violence experiences and PEP cascades(knowledge, access, and utilization), which requires further investigation in future trials. Second, although we found a statistically significant association between FSI and violent experiences, our cross-sectional design precluded causal inference. Future studies should use more rigorous designs and causal inference methods to ascertain the direct effects of FSI on subsequent experiences of violence. Third, responses to our single and self-reported measures for FSI and violence experiences may have been affected by recall bias. Although our study used measures employed by prior relevant studies [13, 40, 43], future studies should use validated multiple-item scales to mitigate the effects of recall bias on the findings and more fully capture FSI- and violence-related data. These measures should also be sequenced to allow for the timing of FSI and violence perpetration or victimization to be recorded, which would allow researchers to model how FSI timing is associated with current or future experiences of violence.

## Conclusion

FSI may contribute to a host of detrimental physical and mental health outcomes in the later years. Disproportionately high rates of FSI (often occurring before survivors were 15 years old) among displaced AGYW living in the informal urban settlements of Kampala, Uganda, highlight the need for sexual violence prevention programs in humanitarian contexts to include girls under 15 years old. We also found significant associations between FSI and experiences of partner and non-partner sexual and physical violence that increased AGYW’s vulnerability to HIV, underscoring the complex and multifaceted nature of sexual violence in humanitarian settings. Our study reveals a concerning landscape of sexual violence and inadequate PEP knowledge, access, and utilization among urban displaced AGYW, highlighting critical gaps in youth sexual health education and service provision, potentially increasing the risk of HIV transmission in an already vulnerable group.
